# Supplementary material for: Learning and memory in the orange head cockroach (Eublaberus posticus)
Source: PLoS One. 2022 Aug 22;17(8):e0272598. doi: 10.1371/journal.pone.0272598 (PMC9394846; doi:10.1371/journal.pone.0272598)
Supplement: S1 File — (PDF) [file pone.0272598.s001.pdf]

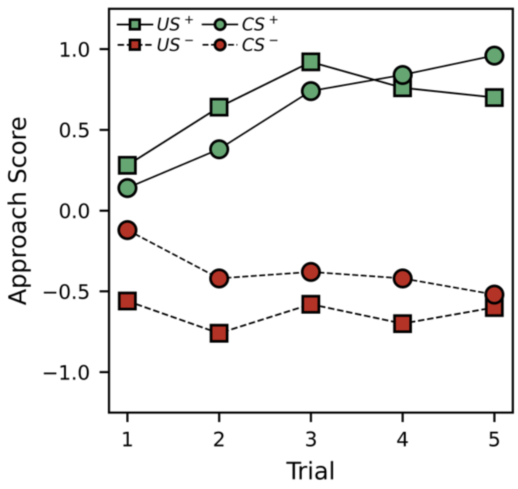

Figure S1. Experiment 2 average approach scores in response to the US and CS divided trial type, during the conditioning trials. Positive approach scores indicate moving or rotating toward a stimulus, negative approach scores indicate moving or rotating away from a stimulus, and zero approach scores indicate no change.

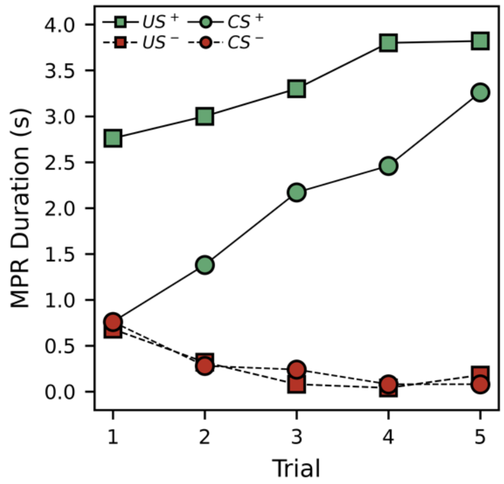

Figure S2. Experiment 2 average MPR duration in response to the US and CS, divided trial type, during the conditioning trials.

Table S1. *Experiment 2 Conditioning US Approach Scores*

Parameter	Estimate	Standard Error	95% Confidence Intervals		*p*-value
Appetitive	0.372	0.114	0.149	0.595	0.001
Aversive	-0.634	0.110	-0.849	-0.419	0.000
Appetitive * Trial	0.096	0.031	0.035	0.157	0.002
Aversive * Trial	-0.002	0.033	-0.067	0.063	0.952
**Pairwise Comparison**			**Difference**	***z*-score**	***p*-value**
Appetitive vs. Aversive			1.006	6.364	0.000
Appetitive * Trial vs. Aversive * Trial			0.098	2.143	0.032

Table S2. *Experiment 2 Conditioning CS Approach Scores*

Parameter	Estimate	Standard Error	95% Confidence Intervals		*p*-value
Appetitive	-0.018	0.119	-0.251	0.215	0.879
Aversive	-0.132	0.120	-0.368	0.104	0.272
Appetitive * Trial	0.210	0.026	0.159	0.261	0.000
Aversive * Trial	-0.080	0.030	-0.140	-0.020	0.009
**Pairwise Comparison**			**Difference**	***z*-score**	***p*-value**
Appetitive vs. Aversive			0.114	0.675	0.500
Appetitive * Trial vs. Aversive * Trial			0.290	7.210	0.000

Table S3. *Experiment 2 Conditioning US MPR Duration*

Parameter	Estimate	Standard Error	95% Confidence Intervals		*p*-value
Appetitive	2.460	0.238	1.993	2.927	0.000
Aversive	0.644	0.164	0.322	0.966	0.000
Appetitive * Trial	0.292	0.055	0.185	0.399	0.000
Aversive * Trial	-0.128	0.040	-0.206	-0.050	0.001
**Pairwise Comparison**			**Difference**	***z*-score**	***p*-value**
Appetitive vs. Aversive			1.816	6.276	0.000
Appetitive * Trial vs. Aversive * Trial			0.420	6.204	0.000

Table S4. *Experiment 2 Conditioning CS MPR Duration*

Parameter	Estimate	Standard Error	95% Confidence Intervals		*p*-value
Appetitive	0.182	0.200	-0.210	0.574	0.363
Aversive	0.756	0.159	0.444	1.068	0.000
Appetitive * Trial	0.608	0.050	0.510	0.706	0.000
Aversive * Trial	-0.156	0.041	-0.237	-0.075	0.000
**Pairwise Comparison**			**Difference**	***z*-score**	***p*-value**
Appetitive vs. Aversive			-0.574	-2.245	0.025
Appetitive * Trial vs. Aversive * Trial			0.764	11.778	0.000

Table S5. *Experiment 2 Preference MPR Latency*

Parameter	Estimate	Standard Error	95% Confidence Intervals		*p*-value
Appetitive	9.239	1.546	6.208	12.269	0.000
Aversive	18.111	1.871	14.444	21.779	0.000
Appetitive * Time	0.006	0.003	0.000	0.011	0.055
Aversive * Time	-0.004	0.002	-0.008	0.000	0.029
**Pairwise Comparison**			**Difference**	***z*-score**	***p*-value**
Appetitive vs. Aversive			-8.872	-3.655	0.000
Appetitive * Time vs Aversive * Time			-0.010	2.773	0.006

Note. Null values of latency were replaced with the mean of latency.
