# Supplementary material for: Learning and memory in the orange head cockroach (Eublaberus posticus)
Source: PLoS One. 2022 Aug 22;17(8):e0272598. doi: 10.1371/journal.pone.0272598 (PMC9394846; doi:10.1371/journal.pone.0272598)
Supplement: Attachment — Submitted filename: Review Manuscript PONE-D-22-06736.docx [file pone.0272598.s003.docx]

Review Manuscript PONE-D-22-06736

The manuscript nicely describes experiments investigating learning and memory in the orange head cockroach. The authors describe the advantages of this species compared to other cockroach species well and have a good knowledge of the existing prominent work in insect olfactory learning overall.

Experiments and analyses are well described and easy to understand.

I only have the following minor points:

- Line 119: Since the apparatus was not described before in another publication which can be referred to, a figure with a graph of the apparatus and the different chambers would be nice
- Line 141: if I understand correctly, dye was applied to equal the color for the appetitive and aversive solutions?
- Line 282: add **for** before unpaired trials
- Line 326: I find the sample size quite different. It would be nicer for comparison to have approximately equal sample size
- Line 400: correct **proboscis**
- Line 422: add **of** before the experiments
