# Supplementary material for: Learning and memory in the orange head cockroach (Eublaberus posticus)
Source: PLoS One. 2022 Aug 22;17(8):e0272598. doi: 10.1371/journal.pone.0272598 (PMC9394846; doi:10.1371/journal.pone.0272598)
Supplement: Attachment — Submitted filename: Response to Reviewers.docx [file pone.0272598.s004.docx]

**Comments from the editor**

We have now received two reviews of your submitted paper, and both reviewers support the publication of your study and suggest several corrections and changes. Specifically, there are no concerns with your actual experiments, except for the unequal sample size, which you should address. Reviewer #1 has concerns with your rational advocating for Eublaberus posticus cockroaches. While you point out that these cockroaches are less likely to escape compared to Periplaneta americana and Blattella germanica, there are other species such as in the genera Blaberus or Gromphadorhina, which are very well studied, and which you should discuss and include in your argument. While promoting Eublaberus posticus is fine, you should discuss and compare the overall advantages and disadvantages of different cockroach species for research and education. You should take into account all the reviewers' suggestions and include a figure of your experimental setup, which is difficult to envision from your methods description.

*We appreciate the efforts of the editor and reviewer. We have made several revisions described in the following sections. We believe we have addressed all the suggestions, and the manuscript is improved as a result.*

**Comments from reviewer #1**

The authors utilized learning paradigms and implemented the behavior with the orange
orange head cockroach (*Eublaberus posticus*), which has not been demonstrated. Further, the authors state that the goal of this project is to use the orange head cockroach as a model organism for behavioral research. The title and stating the goal definitely sets the stage, and offer a reason as to "why" the orange head cockroach is advantageous as a model organism. I was expecting a myriad of early literature (not cited) elucidating the utilization of cockroaches for learning and memory. I would make sure that if the intro sets the stage, it must be followed by advantages / disadvantages. Early on, there is mention about escape. How so? Is the claim that cockroaches are useful model organisms? is the claim that orange head cockroach is a better species over other cockroaches? I am not convinced. I like how the intro sets up the stage, yet it is never explicitly mentioned. I would build upon the initial idea about the use of invertebrates as models organisms to study specific aspects of disease or health are available. There is lacking how the link to the orange head cockroach is important and how so over other cockroaches? Are there benefits ? (list them) over others that show impressive behavioral abilities.

*The reviewer has concerns about the rationale of use of the orange head cockroach, and requests additional discussion of the benefits of this species. Discussion of species selection is a topic we enjoy, but this particular discussion can be challenging as there is so little information on cockroach learning outside of Periplaneta americana and Blattella germanica, as we stated in our paper.*

*First, we would like to first clarify that our claims are 1) that cockroaches may be useful complements to existing insect models, and 2) that the orange head cockroach is one practical choice that may be a good fit for many laboratories. They may be especially good choices for those concerned about the practical considerations of working with a domiciliary pest. At my institution, for example, we do not believe we would receive the same level of support and interest, from administration and students alike, if we were working with a pest species. However, while we do claim the orange head cockroach is a good alternative, we do not intend to claim it is the only alternative, or the only species that should be investigated.*

*Second, we would like to note that there are several other articles published in PLoS ONE on cockroach conditioning that do not include a substantial section of subject choice, even one that uses a less common species. We also provide over 20 varied citations on the general use of cockroaches in learning and neurophysiology work. We feel that our introduction is consistent with the level of detail included in these articles and provides a good background for the reader.*

*Watanabe H, Mizunami M (2007) Pavlov's Cockroach: Classical Conditioning of Salivation in an Insect. PLoS ONE 2(6): e529. https://doi.org/10.1371/journal.pone.0000529*

*Matsumoto Y, Hirashima D, Terao K, Mizunami M (2013) Roles of NO Signaling in Long-Term Memory Formation in Visual Learning in an Insect. PLoS ONE 8(7): e68538. https://doi.org/10.1371/journal.pone.0068538*

*Garren MV, Sexauer SB, Page TL (2013) Effect of Circadian Phase on Memory Acquisition and Recall: Operant Conditioning vs. Classical Conditioning. PLoS ONE 8(3): e58693. https://doi.org/10.1371/journal.pone.0058693*

*Finally, to improve our discussion of this topic in the paper, we expanded the introduction from four paragraphs to six paragraphs. We emphasized that Eublaberus posticus is one *potential* alternative, but not necessarily the only alternative to pest species. We mentioned three other well-known alternatives that readers would be likely to consider (Blaberus discoidalis, Blaptica dubia, and Gromphadorhina portentosa) and suggested that our primary reason to focus on Eublaberus posticus is due to its strong feeding behavior. A major benefit of this discussion is that some behaviorally inclined readers that are not necessarily entomologically inclined may be made aware of options, where they may otherwise think cockroach refers exclusively to pests and hissing cockroaches. Our revised paragraphs are below:*

*Most research on cockroach behavior, including the preceding citations, has been on Periplaneta americana or Blattella germanica, two well-known domiciliary pests [16]. As both species are excellent climbers, and P. americana is also able to fly, some laboratories may have concerns about housing these potential pests. We believe the orange head cockroach, Eublaberus posticus, may be a good alternative for laboratories that are concerned about the escape and infestation potential of pest species. E. posticus is a large Central and South American species that, unlike P. americana and Blattella germanica, would have difficulty surviving outside of a tropical environment. Additionally, E. posticus is unable to fly or climb smooth surfaces, making escapes rare and simplifying apparatus design. While there are a few studies on the social behavior of E. posticus [51, 52], we are only familiar with a single paper on learning in this species; a series of habituation experiments conducted by our laboratory [53].*

*We believe that E. posticus is an ideal alternative to potential pest species, though not necessarily the only alternative. Closely related species in the genera Blaberus and Eublaberus may also be good candidates as they are tropical species that are generally unable to fly or climb smooth surfaces, with Blaberus discoidalis being another well-known, easy to acquire species. Blaptica dubia is another popular and related species that shares these practical traits. The common hissing cockroach, Gromphadorhina portentosa, is perhaps the best-known non-pest species. However, unlike E. posticous, they are excellent climbers [15]. More importantly, in pilot research, we have found their appetite to be inconsistent. While G. portentosa has fascinating vocal and social behavior, we have found their weak appetite makes traditional food-related conditioning procedures challenging. We selected E. posticous over other well-known species with similar characteristics, such as Blaberus discoidalis and Blaptica dubia, as E. posticus is known to have a voracious appetite, even predating on smaller insects [15]. Feeding reliability is an important component of many associative conditioning procedures, and the need for reliable feeding behavior was especially salient for us after pilot work with G. portentosa.*

*In this paper, we investigate associative learning and memory in E. posticus in two experiments. In our first experiment, we investigate the ability of E. posticus to learn to associate an arbitrary conditioned stimulus odor with either an appetitive food solution or an aversive saltwater solution. In our second experiment, we repeat the conditioning methods of the first experiment, but also test for preferences to conditioned odors 15 to 1,215 minutes after conditioning to explore the extent that associative memories are retained.*

There is a disconnect, at least in my mind, since it is not spelled out by the authors in my view. It was not clear why the experimental design and methods were chosen (odor, other than mentioning associative learning) and why and how these are suited for the orange head cockroach? This needs to be developed.

*The general methods were modeled after other cockroach and bee procedures. We provide a few citations for this in the paper:*

*To collect motivated subjects, we placed an inaccessible cup of food inside the colony and collected only individuals that immediately approached the cup. This method is modeled after other techniques to ensure collection of motivated subjects found in cockroach and bee conditioning research [24, 54, 55].*

*The individual chambers were sized to allow subjects to freely move inside the container [55-58], rather than restrain the subjects [24, 31, 54], as while the restrained methods are known to work well with honey bees, other species may perform poorly when restrained [56].*

*We developed this solution based on the use of sucrose solutions in cockroach and bee research [24, 34, 54, 55, 58], as well as pilot research suggesting the inclusion of dog food was important.*

*We selected this US^-^ based on other research with cockroaches [55], and because we found this ratio of NaCl and water was aversive but did not inhibit feeding from other solutions in pilot experiments.*

*We selected these odors as our pilot research suggested they were neutral, unlike vanilla and peppermint which may be innately appetitive and aversive, respectively, to some species of cockroach [55].*

*The solution was lightly touched to the antennae unless the subject immediately made contact with its mouthparts. This solution presentation method is common to both cockroach and bee conditioning procedures [24, 36, 54].*

*We also recorded maxillary palp extension response (MPR) during both the conditioning trials and the preference test as an analog to the proboscis extension response (PER) commonly used to study bee learning [24].*

*We added an additional statement to support our use of odors as CS:*

*We used odors as stimuli due to the prevalence of olfactory conditioning in the insect literature including several experiments with cockroaches [6, 8, 9, 32-36, 54-57].*

*We also added an additional statement to support our general conditioning methods for the paired and unpaired groups in experiment 1.*

*The general methods for both groups were based on established honey bee procedures [24, 54], as well as recent cockroach work [32, 33, 36, 55].*

*Unfortunately, while honey bee conditioning methods have been well-investigated and standardized to an extent, this is still a fairly new area of research for cockroaches. As this is the first associative learning procedure with this species, there is much we simply do not yet know about the ideal methods. However, we believe that the support we have provided is a sufficient for the paper, and a helpful guide for future work.*

**Comments from reviewer #2**

The manuscript nicely describes experiments investigating learning and memory in the orange head cockroach. The authors describe the advantages of this species compared to other cockroach species well and have a good knowledge of the existing prominent work in insect olfactory learning overall. Experiments and analyses are well described and easy to understand.
I only have the following minor points:

• Line 119: Since the apparatus was not described before in another publication which can be referred to, a figure with a graph of the apparatus and the different chambers would be nice

*Thank you for this suggestion. We included an illustration of the larger filming unit, and the individual experiment chambers that house the subjects.*

***Fig 1. Apparatus diagram.*** *(A) Filming unit containing four clear plastic experiment chambers. Three internal lights were used to supplement room lighting. Mirrors were used to film the ventral side of subjects. (B) Top view of one experiment chamber. Six holes in the top of the chambers permitted stimulus administration.*

• Line 141: if I understand correctly, dye was applied to equal the color for the appetitive and aversive solutions?

*This is correct. The appetitive solution had a slight color to it, so the dye was used to reduce the possibility the subjects learned any visual associations. The preference tests after conditioning was complete did indicate the subjects learned the olfactory associations, but we also wanted to minimize any visual cues that were given. We added a note to clarify this further at the end of this paragraph.*

*Dying both solutions served to reduce the possibility the subjects could learn visual distinctions between the appetitive and aversive solutions.*

• Line 282: add for before unpaired trials

*We made this correction.*

• Line 326: I find the sample size quite different. It would be nicer for comparison to have approximately equal sample size

*We apologize, as an error on our part introduced some confusion. The figure, now Fig 4, shows the average MPR duration and latency for the preference test of experiment 1. We noted that averaged values exclude non-responders. This was not accurate. The average durations values include data from all 64 subjects; a 0 is used if the subject responded for 0 seconds. Only the averaged latency values exclude non-responders. As there are multiple options for dealing with the latency of non-responders (such as replacing the data with the mean or maximum values of latency), we elected to simply not include non-responders in the latency graph, as it appeared to be the best option for a visual display of data. We then reported the number of animals that responded for the sake of transparency. We edited the caption for Fig 4 and Fig 6 to correct and further clarify this point.*

***Fig 4. Experiment 1 average MPR duration and latency for the paired and unpaired groups during the preference test.*** *The number of subjects responding to the CS^+^, CS^-^, CS^u^ and CS^n^ were 25, 10, 13, and 20, respectively. Averaged latency values exclude non-responders.*

***Fig 6.*** ***Experiment 2 average MPR latency to respond to the CS^+^ and CS^-^ during the preference test.*** *For the CS^+^, the number of subjects that responded in the 15-, 24-, 135-, 405- and 1,215-minute delay groups were 7, 9, 8, 6 and 1, respectively. For the CS^-^, the number of subjects that responded were 2, 1, 2, 0 and 0.*

*We also want to clarify that the difference in number of animals that respond is a result, not a methodological issue. As the animals are free to respond, or not respond, it is not possible to ensure an equal number of responders without collecting data from additional subjects, and even then, subjects may not respond in all conditions. Additionally, if more data is collected to balance the number of responses for the final preference test, the group sizes then become unbalanced for the conditioning procedure. All statistical analysis are able to deal with this issue by using a duration of 0 for non-responders, a replacing a null latency with mean latency for non-responders, or by analyzing probability of response.*

• Line 400: correct proboscis

*We made this correction.*

• Line 422: add of before the experiments

*We made this correction.*
